# Treatment of idiopathic pulmonary fibrosis: a network meta-analysis

**DOI:** 10.1186/s12916-016-0558-x

**Published:** 2016-02-03

**Authors:** Bram Rochwerg, Binod Neupane, Yuan Zhang, Carlos Cuello Garcia, Ganesh Raghu, Luca Richeldi, Jan Brozek, Joseph Beyene, Holger Schünemann

**Affiliations:** Department of Medicine, Division of Critical Care, McMaster University, 1200 Main St W, L8S 4L8 Hamilton, ON Canada; Department of Clinical Epidemiology & Biostatistics, McMaster University, Hamilton, ON Canada; MacGRADE Centre, McMaster University, Hamilton, ON Canada; Tecnologico de Monterrey, School of Medicine, Monterrey, Mexico; Department of Medicine (Division of Pulmonary and Critical Care Medicine), University of Washington Seattle, Seattle, WA USA; National Institute for Health Research Southampton Respiratory Biomedical Research Unit and Clinical and Experimental Sciences, University of Southampton, Southampton, UK

**Keywords:** Idiopathic pulmonary fibrosis, Network meta-analysis, Systematic review, Treatment

## Abstract

**Background:**

Idiopathic pulmonary fibrosis (IPF) is an interstitial lung disease associated with high morbidity and mortality. Effective treatments for IPF are limited. Several recent studies have investigated novel therapeutic agents for IPF, but very few have addressed their comparative benefits and harms.

**Methods:**

We performed a Bayesian network meta-analysis (NMA) to assess the effects of different treatments for IPF on mortality and serious adverse events (SAEs). We searched MEDLINE and EMBASE for randomized controlled trials (RCTs) up to August 2015. The Grading of Recommendations Assessment, Development and Evaluation (GRADE) approach served to assess the certainty in the evidence of direct and indirect estimates. We calculated the surface under the cumulative ranking curve (SUCRA) for each treatment.

We included parallel group RCTs, including factorial designs, but excluded quasi-randomized and cross-over trials. Studies were only included if they involved adult (≥18 years of age) patients with IPF as defined by the 2011 criteria and examined one of the 10 interventions of interest (ambrisentan, bosentan, imatinib, macitentan, N-acetylcysteine, nintedanib, pirfenidone, sildenafil, prednisone/azathioprine/N-acetylcysteine triple therapy, and vitamin K antagonist).

**Results:**

A total of 19 RCTs (5,694 patients) comparing 10 different interventions with placebo and an average follow-up period of 1 year fulfilled the inclusion criteria. SUCRA analysis suggests nintedanib, pirfenidone, and sildenafil are the three treatments with the highest probability of reducing mortality in IPF. Indirect comparison showed no significant difference in mortality between pirfenidone and nintedanib (NMA OR, 1.05; 95 % CrI, 0.45–2.78, moderate certainty of evidence), pirenidone and sildenafil (NMA OR, 2.26; 95 % CrI, 0.44–13.17, low certainty of evidence), or nintedanib and sildenafil (NMA OR 2.40; 95 % CrI, 0.47–14.66, low certainty of evidence). Sildenafil, pirfenidone, and nintedanib were ranked second, fourth, and sixth out of 10 for SAEs.

**Conclusion:**

In the absence of direct comparisons between treatment interventions, this NMA suggests that treatment with nintedanib, pirfenidone, and sildenafil extends survival in patients with IPF. The SAEs of these agents are similar to the other interventions and include mostly dermatologic and gastrointestinal manifestations. Head-to-head comparisons need to confirm these findings.

## Background

Idiopathic pulmonary fibrosis (IPF) is a progressive interstitial pneumonia of unknown cause that usually affects older adults and is associated with a median survival of 3–5 years after the time of diagnosis [1, 2]. The diagnostic criteria, clinical characteristics, and natural course of the disease have been well defined in recent evidence-based guidelines for the diagnosis and management of IPF [2]. IPF manifests with worsening dyspnea and a high degree of morbidity experienced by patients [1]. Patients with IPF often experience a step-wise decline in pulmonary function test (PFT) parameters and clinical symptoms, and acute exacerbations are associated with increased mortality. Until recently, despite an increasing number of clinical trials, no intervention, other than lung transplantation, had demonstrated an enhanced survival in patients with IPF [2]. However, recent large scale randomized controlled trials (RCTs) of a few novel agents have demonstrated a decreased rate of disease progression as measured by forced vital capacity (FVC) in well-defined patients with IPF [3–5].

The choice of first line treatment is best addressed by direct comparisons of treatment regimens in high quality studies, but such studies do not yet exist for IPF. Previous systematic reviews and meta-analyses have relied on direct comparisons [6, 7]. A recently published multiple comparison analysis showed the potential benefit of nintedanib and pirfenidone compared to other treatment interventions [8]. Further, based on indirect comparison, results suggested that nintedanib might be superior to pirfenidone in slowing the rate of FVC decline [8]. This review had limitations as it focused on only a select number of interventions (three in total, including N-acetylcysteine monotherapy, nintedanib, and pirfenidone), which limited the evidence to a fraction of that available. More importantly, it focused on the outcome of FVC, a correlate for survival [9], and, due to its variable reporting across included studies (including FVC outcome measures such as percent change, percent predicted, volume change, etc.), the analysis relied on standardized mean differences, which limit application in decision-making [8].

We performed a multiple treatment comparison based on a network meta-analysis considering both direct and indirect comparisons of 10 treatment interventions that have been tested in RCT of patients with well-defined IPF. We focused on mortality and SAEs, as these outcomes are clinically relevant and meaningful to patients.

## Methods

We conducted this systematic review to inform the clinical practice guidelines for the pharmacologic treatment of patients with IPF sponsored by the American Thoracic Society, European Respiratory Society, Japanese Respiratory Society, and the Asociacion Latinoamericana de Torax Society [10]. This multiple comparison network meta-analysis (NMA) followed the guideline development process and was independent of it in that the results of this NMA were not available for the formulation of the guidelines.

For the previous guideline document, published in 2011, we had performed an evidence synthesis of treatment interventions for IPF [2]. For this NMA, we updated the 2010 review and searched for more recent publications only. We utilized the Ovid platform to search MEDLINE, EMBASE, Cochrane Registry of Controlled Trials, Health Technology Assessment, and the Database of Abstracts of Reviews of Affects for the period of May 2010 (the date since the last search) through August 2015 (see Appendix for search strategy). Reviewers (BR, CC, YZ) contacted experts and reviewed previous meta-analyses for additional articles.

Three reviewers (BR, CC, YZ) screened the titles and abstracts in duplicate to determine potential eligibility and entries identified by any reviewer proceeded to the full-text eligibility review. Pre-tested eligibility forms were used for full text review, which was also performed in duplicate, with a third adjudicator (HJS) helping to reach consensus in situations of disagreement. We included parallel group RCTs, including factorial designs, but excluded quasi-randomized and cross-over trials. No language restrictions were applied. Studies were only included if they involved adult (≥18 years of age) patients with IPF as defined by the 2011 criteria [2]. Studies that included patients with other confounding respiratory conditions and idiopathic interstitial idiopathic pneumonia other than IPF were excluded. Studies had to examine treatment with one of the 10 identified interventions of interest included in the guideline update (ambrisentan, bosentan, imatinib, macitentan, N-acetylcysteine, nintedanib, pirfenidone, sildenafil, prednisone/azathioprine/N-acetylcysteine triple therapy, and vitamin K antagonist) compared with one of the other interventions or placebo. We focused on mortality and rates of severe adverse events (SAEs) as data for these outcomes were considered important to patients and widely available across RCT.

Data was abstracted in duplicate and authors of primary publications were contacted when required for missing or unclear information. Individual study risk of bias (RoB) was assessed independently and in duplicate. Reviewers assessed RoB using a tool modified from that recommended by the Cochrane Collaboration [11, 12]. For each included study we provided a judgment of ‘low RoB’, ‘probably low RoB’, ‘probably high RoB’, or ‘high RoB’ for each of the following items: randomization sequence generation, randomization concealment, blinding, incomplete data, selective reporting, and other bias (including lack of intention-to-treat analysis). The overall rating of RoB for each individual study was the lowest of the ratings for any of the RoB criteria.

Heterogeneity in treatment effects was evaluated by estimating the variance between studies, and through Cochrane Q-test and I^2^ [13–15] when at least two studies were available for each pairwise comparison. Under a Bayesian framework, we used a Markov Chain Monte Carlo algorithm to carry out a random effects NMA, where binomial distribution was used for the number of mortality or SAE events within studies. Multiple treatment NMAs allows for the combination of direct and indirect evidence into a combined overall point estimate. We also performed a post-hoc subgroup analysis excluding two trials with follow-up of only 6 months duration, both of which examined sildenafil treatment (with placebo).

We report odds ratios (OR) and their corresponding 95 % credibility intervals (CrI), which are the Bayesian analog of the 95 % confidence intervals [16]. The ORs reported are relative effects of IPF treatments in reducing mortality or SAEs in IPF patients within (an average of) 1 year. Vague (non-informative) priors were used for model parameters and convergence was assessed using Brooks Gelman Rubin plots [17], as well as trace and time-series plots. Goodness-of-fit was evaluated using the mean residual deviance and the surface under the cumulative ranking curve (SUCRA) was employed to rank the treatments [18]. SUCRA is generated based on cumulative probability plots, an intervention which always ranks first would have a SUCRA value of one, whereas one that always ranks last would have a value of zero. We also generated clustered ranking plot of the network based on cluster analysis of SUCRA values for the two outcomes (mortality or SAE). This exploratory plot allows for identification of clusters of treatments that have similar effectiveness and safety profiles [19]. The Bayesian network meta-analysis was conducted using the R statistical package.

The Grading of Recommendations Assessment, Development and Evaluation (GRADE) approach specific to NMA served to assess the certainty in the evidence (quality of evidence) associated with specific comparisons, including direct, indirect, and final network meta-analysis estimates [20]. Our confidence assessment addressed the RoB (in individual studies), imprecision, inconsistency (heterogeneity in estimates of effect across studies), indirectness (related to the question or due to intransitivity), and publication bias [20]. Incoherence assessment was not needed in this analysis as all estimates included only direct (interventions vs. placebo) or only indirect evidence (for all other comparisons). For direct comparisons, the starting point for certainty in estimates was ‘high’ and for indirect comparisons we lowered the starting certainty to ‘moderate’. The certainty in indirect estimates was inferred from examination of the connecting network loops associated with the particular comparison. The certainty rating chosen was the lowest of the direct estimates contributing to the indirect comparison. The judgment of precision was based on the credible interval around the point estimate from the indirect comparison. Publication bias could not be formally assessed based on statistical criteria due to the small number of studies included in the direct comparisons. Although the potential for this bias is real given the small number of studies and the for-profit interest, we did not believe this concern was sufficient enough to further downgrade the certainty in the evidence.

## Results

**Fig. 1:**
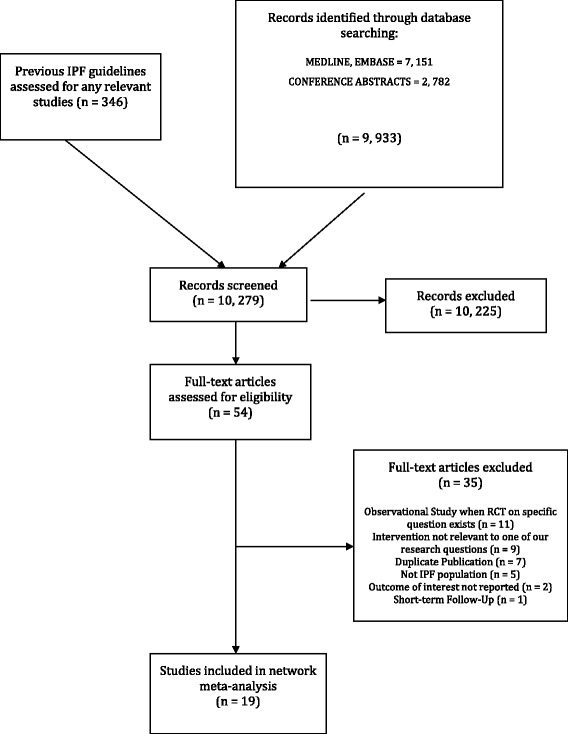
Flow chart of search results

A total of 9,933 titles were identified during the primary search (Fig. 1), and were combined with 346 studies found through screening titles included in the previous iteration of the IPF guidelines. Of these 10,279 references, 10,225 were judged as ineligible on the basis of titles and abstracts, leaving 54 studies for full text review, of which 35 proved ineligible, leaving 19 eligible RCTs that were included in the final analysis [3–5, 21–35].

Table 1 summarizes the characteristics of these 19 RCTs involving 5,694 adults. All trials examined patients diagnosed with IPF according to current international diagnostic criteria [2]. Most trials focused on patients with mild or moderate impairment in PFTs or other clinical parameters used to exclude patients with severe functional impairment as a result of their lung disease.

**Table 1:** Study characteristics

	Number of randomized patients	ATS/ERS/JRS/ALAT clinical criteria used for diagnosis	Pulmonary function tests/blood gas criteria for enrolment	Intervention (all compared to placebo)	Follow-up used for analysis	Risk of bias	Mortality	Severe adverse events	Industry sponsor
Noth 2012 [21]	Multicenter (22) USA n = 146	Yes – no bronchoscopy required	Progressive decline in FVC (>10 % in last year) or DLCO (>15 % in last year)	Warfarin	12 months	High (trial stopped early for harm)	VKA 14/72 Placebo 3/74	21/72 12/74	None
PANTHER 2012 [22]	Multicenter (25) USA n = 155	Yes	FVC >50 % predicted DLCO >30 % predicted	Triple therapy (NAC, azathioprine, predinisone)	12 months	High (trial stopped early for harm)	Triple Rx 8/77 Placebo 1/78	24/77 8/78	None
Jackson 2010 [23]	Single Center USA n = 29	Yes – no bronchoscopy required	FVC 40–90 % predicted DLCO 30–90 % predicted	Sildenafil	6 months	Low	Sildenafil 0/14 Placebo 0/15	0/14 0/15	Pfizer UK
STEP-IPF 2010 [24]	Multicenter (14) USA n = 180	Yes	DLCO <35 % predicted	Sildenafil	6 months	Low	Sildenafil 3/89 Placebo 9/91	13/89 15/91	Pfizer
Homma 2012 [25]	Multicenter (27) Japan n = 88	Yes Also required elevated markers pneumocyte injury (KL-6, surfactant protein A & D)	Partial arterial oxygen concentration >70 mmHg at rest	NAC monotherapy	12 months	Low	NAC 0/44 Placebo 0/46	Not reported	None
Tomioka 2005 [26]	Single Center Japan n = 30	Yes	None	NAC monotherapy	12 months	Low	NAC 2/15 Placebo 2/15	0/15 0/15	None
IPF Network 2014 [3]	Multicenter (25) USA n = 264	Yes	FVC >50 % predicted DLCO >30 % predicted	NAC monotherapy	12 months	Low	NAC 6/133 Placebo 3/131	25/133 20/131	None
IFIGENIA 2005 [36]	Multicenter (36) Europe n = 182	No Histologic or radiologic pattern of UIP with other causes ruled out. Mandatory biopsy in patients <50 years old Mandatory bronchoscopy and duration >3 months	FVC <80 % predicted TLC <90 % predicted DLCO <80 % predicted	NAC monotherapy	12 months	Low	NAC 7/92 Placebo 8/90	Not reported	Zambon
Azuma 2005 [27]	Multicenter (25) Japan n = 108	Yes	None	Pirfenidone	9 months	High (trial stopped early for benefit)	Pirfenidone 0/73 Placebo 1/35	Not reported	Shinogi & Co.
Taniguchi 2010 [29]	Multicenter (73) Japan n = 212	Yes	None	Pirfenidone	12 months	Low	Pirfenidone 3/108 Placebo 4/104	Not reported	None
CAPACITY 2011 [28]	Multicenter (110) Worldwide n = 692	Yes	FVC >50 % predicted DLCO >35 % predicted	Pirfenidone	24 months	Low	Pirfenidone 27/345 Placebo 34/347	113/345 109/347	Intermune
King Jr 2014 [4]	Multicenter (127) Worldwide n = 555	Yes	FVC 50–90 % predicted DLCO 30–90 % predicted FEV1/FVC >80 %	Pirfenidone	12 months	Low	Pirfenidone 11/278 Placebo 20/277	52/278 56/277	Intermune
BUILD-1 2008 [30]	Multicenter (29) Europe, N. America n = 158	Yes	FVC >50 % predicted DLCO >30 % predicted	Bosentan	12 months	Low	Bosentan 3/74 Placebo 3/84	22/74 29/84	Actelion Pharmaceuticals
BUILD-3 2011 [31]	Multicenter (119) Worldwide n = 616	Yes	None	Bosentan	12 months	Low	Bosentan 17/407 Placebo 7/209	129/407 74/209	Actelion Pharmaceuticals
MUSIC 2013 [33]	Multicenter (48) Worldwide n = 178	Yes, with positive biopsy	FVC >50 % predicted DLCO >30 % predicted FEV1/FVC >70 %	Macitentan	12 months	Low	Macitentan 3/119 Placebo 2/59	37/119 20/59	Actelion Pharmaceuticals
ARTEMIS 2013 [32]	Mulitcenter (136) Worldwide n = 492	Yes	None	Ambrisentan	12 months	High (trial stopped early for harm)	Ambrisentan 26/329 Placebo 6/163	73/329 25/163	Gilead Sciences
Daniels 2010 [34]	Multicenter (13) USA & Mexico n = 119	Yes	FVC >55 % predicted DLCO >35 % predicted FEV1/FVC >60 % Progressive decline in FVC (>10 % in last year)	Imatinib	24 months	Low	Imatinib 8/59 Placebo 10/60	18/59 19/60	Novartis Pharmaceuticals
Richeldi 2011 [35]	Multicenter (92) Worldwide n = 428	Yes	FVC >50 % predicted DLCO 30–79 % predicted	Nintedanib	12 months	Low	Nintedanib 25/343 Placebo 9/85	90/343 26/85	Boehringer Ingelheim
INPULSUS 2014 [5]	Multicenter (205) Worldwide n = 1061	Yes	FVC >50 % predicted DLCO 30–79 % predicted	Nintedanib	12 months	Low	Nintedanib 35/638 Placebo 33/423	194/638 127/423	Boehringer Ingelheim

n, Number; VKA, Vitamin K antagonist; FVC, Forced vital capacity; DLCO, Diffusion capacity of lung for carbon monoxide; TLC, Total lung capacity; NAC, N-acetylcysteine

### Mortality

Table 2 shows the NMA and mortality results. The results demonstrate lower mortality associated with sildenafil treatment compared to ambrisentan (NMA OR, 0.12; 95 % CrI, 0.01–0.78, moderate quality of evidence), triple therapy (NMA OR, 0.02; 95 % CrI, 0.01–0.30, moderate quality of evidence), and vitamin K antagonists (VKA) (NMA, OR 0.05; 95 % CrI, 0.01–0.37, moderate certainty in the evidence). Similarly, pirfenidone is associated with a mortality benefit when compared to ambrisentan (NMA OR, 0.28; 95 % CrI, 0.07–0.93, moderate certainty in the evidence), triple therapy (NMA OR, 0.05; 95 % CrI, 0.01–0.44, moderate certainty in the evidence), and VKA (NMA OR, 0.10; 95 % CrI, 0.02–0.47, moderate certainty in the evidence). Nintedanib is beneficial in terms of mortality when compared to only triple therapy (NMA OR, 0.05; 95 % CrI, 0.01–0.49, moderate certainty in the evidence) and VKA (NMA OR, 0.11; 95 % CrI, 0.02–0.54, moderate certainty in the evidence).

**Table 2:**
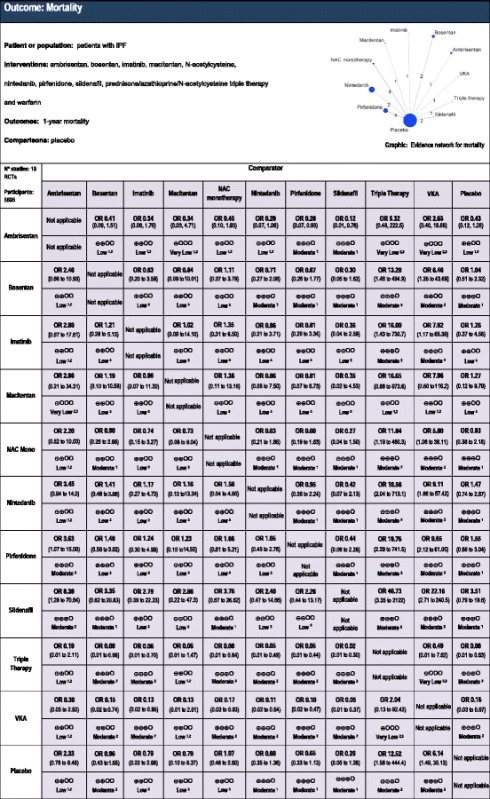
Estimates of effects (with 95 % credible intervals) and confidence ratings for comparisons of therapeutic agents for the treatment of idiopathic pulmonary fibrosis (IPF) on the outcome mortality

OR, Odds ratio

^1^ Certainty lowered for imprecision

^2^ Certainty lowered for individual study risk of bias

^3^ Certainty lowered two levels for imprecision

GRADE Working Group grades of evidence – High quality: Further research is very unlikely to change our confidence in the estimate of effect; Moderate quality: Further research is likely to have an important impact on our confidence in the estimate of effect and may change the estimate; Low quality: Further research is very likely to have an important impact on our confidence in the estimate of effect and is likely to change the estimate; Very low quality: We are very uncertain about the estimate

We found no significant difference when comparing sildenafil to pirfenidone (NMA OR, 0.44; 95 % CrI, 0.08–2.28, moderate certainty in the evidence) or nintedanib (NMA OR, 0.42; 95 % CrI, 0.07–2.13, moderate certainty in the evidence), or when comparing pirfenidone to nintedanib (NMA OR, 0.95; 95 % CrI, 0.36–2.24, moderate certainty in the evidence). Triple therapy is significantly worse than most interventions including imatinib (NMA OR, 16.00; 95 % CrI, 1.43–730.7, moderate certainty in the evidence), NAC monotherapy (NMA OR, 11.84; 95 % CrI, 1.19–480.3, moderate certainty in the evidence), and placebo (NMA OR, 12.52; 95 % CrI, 1.58–444.4, moderate certainty in the evidence), in addition to those listed above. VKA also was associated with a higher mortality compared with imatinib (NMA OR, 7.92; 95 % CrI, 1.17–65.39, moderate certainty in the evidence), NAC monotherapy (NMA OR, 5.80; 95 % CrI, 1.08–38.11, moderate certainty in the evidence), bosentan (NMA OR, 6.46; 95 % CrI, 1.35–43.69, moderate certainty in the evidence), and placebo (NMA OR, 6.14; 95 % CrI, 1.49–35.13, moderate certainty in the evidence) in addition to those listed above.

SUCRA analysis (Table 3) suggested nintedanib, pirfenidone, and sildenafil as the three treatments with the highest probability of reducing mortality in IPF. Subgroup analysis, excluding two trials of sildenafil with only 6-month follow-up, showed nintedanib and pirfenidone to be the two treatments with the highest probability of being efficacious compared with other included interventions.

**Table 3:** Surface under the cumulative ranking curve (SUCRA) data for the outcomes of mortality and severe adverse events

a. SUCRA rankings for mortality	
Treatment	SUCRA
Sildenafil	0.913
Pirfenidone	0.749
Nintedanib	0.719
Imatinib	0.624
Macitentan	0.604
Bosentan	0.533
Placebo	0.490
NAC monotherapy	0.483
Ambrisentan	0.238
VKA	0.097
Triple therapy	0.050
b. SUCRA rankings for severe adverse events	
Treatment	SUCRA
Bosentan	0.756
Sildenafil	0.692
Macitentan	0.684
Nintedanib	0.638
Imatinib	0.627
Pirfenidone	0.597
Placebo	0.587
NAC monotherapy	0.413
Ambrisentan	0.279
VKA	0.190
Triple therapy	0.038

SUCRA, Surface under cumulative ranking curve

### Severe adverse events (SAEs)

Four of the 19 trials did not report SAEs and were therefore not included in this analysis [25, 27, 29, 36]. Table 4 shows the NMA and SAE results. Triple therapy showed a significant increase in SAEs compared with bosentan (NMA OR, 4.94; 95 % CrI, 1.52–17.70, low certainty in the evidence), imatinib (NMA OR, 4.35; 95 % CrI, 1.05–20.05, low certainty in the evidence), macitentan (NMA OR, 4.74; 95 % CrI, 1.18–20.63, low certainty in the evidence), nintedanib (NMA OR, 4.35; 95 % CrI, 1.36–15.47, low certainty in the evidence), pirfenidone (NMA OR, 4.17; 95 % CrI, 1.29–14.51, low certainty in the evidence), sildenafil (NMA OR, 4.91; 95 % CrI, 1.11–22.48, low certainty in the evidence), and placebo (NMA OR, 4.15; 95 % CrI, 1.43–12.88, low certainty in the evidence).

**Table 4:**
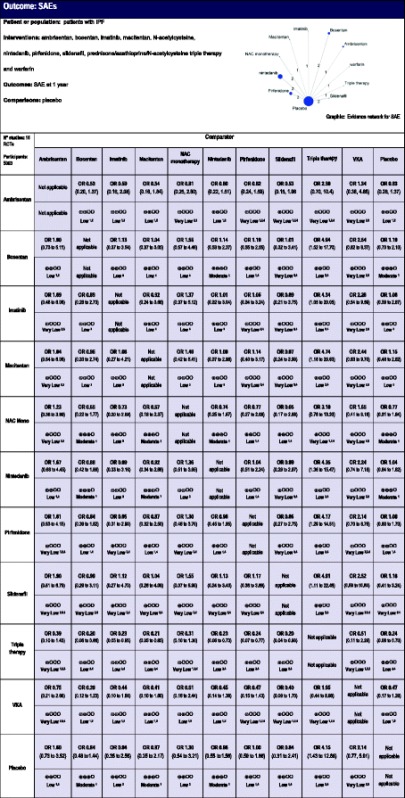
Estimates of effects (with 95 % credible intervals) and confidence ratings for comparisons of therapeutic agents for the treatment of idiopathic pulmonary fibrosis (IPF) on the outcome severe adverse events (SAEs)

OR, Odds ratio

^1^ Certainty lowered for imprecision

^2^ Certainty lowered for individual study risk of bias

^3^ Certainty lowered two levels for imprecision

^4^ Certainty lowered for indirectness

GRADE Working Group grades of evidence – High quality: Further research is very unlikely to change our confidence in the estimate of effect; Moderate quality: Further research is likely to have an important impact on our confidence in the estimate of effect and may change the estimate; Low quality: Further research is very likely to have an important impact on our confidence in the estimate of effect and is likely to change the estimate; Very low quality: We are very uncertain about the estimate

SUCRA analysis (Table 4) suggested that bosentan, macitentan, and sildenafil had the lowest risk of SAEs. Nintedanib and pirfenidone were ranked fourth and sixth, respectively. VKA and triple therapy were the two lowest ranked interventions with the highest probability of causing SAEs. Subgroup analysis, excluding two trials of sildenafil with only 6-month follow-up, demonstrated very similar results.

### SUCRA cluster

**Fig. 2:**
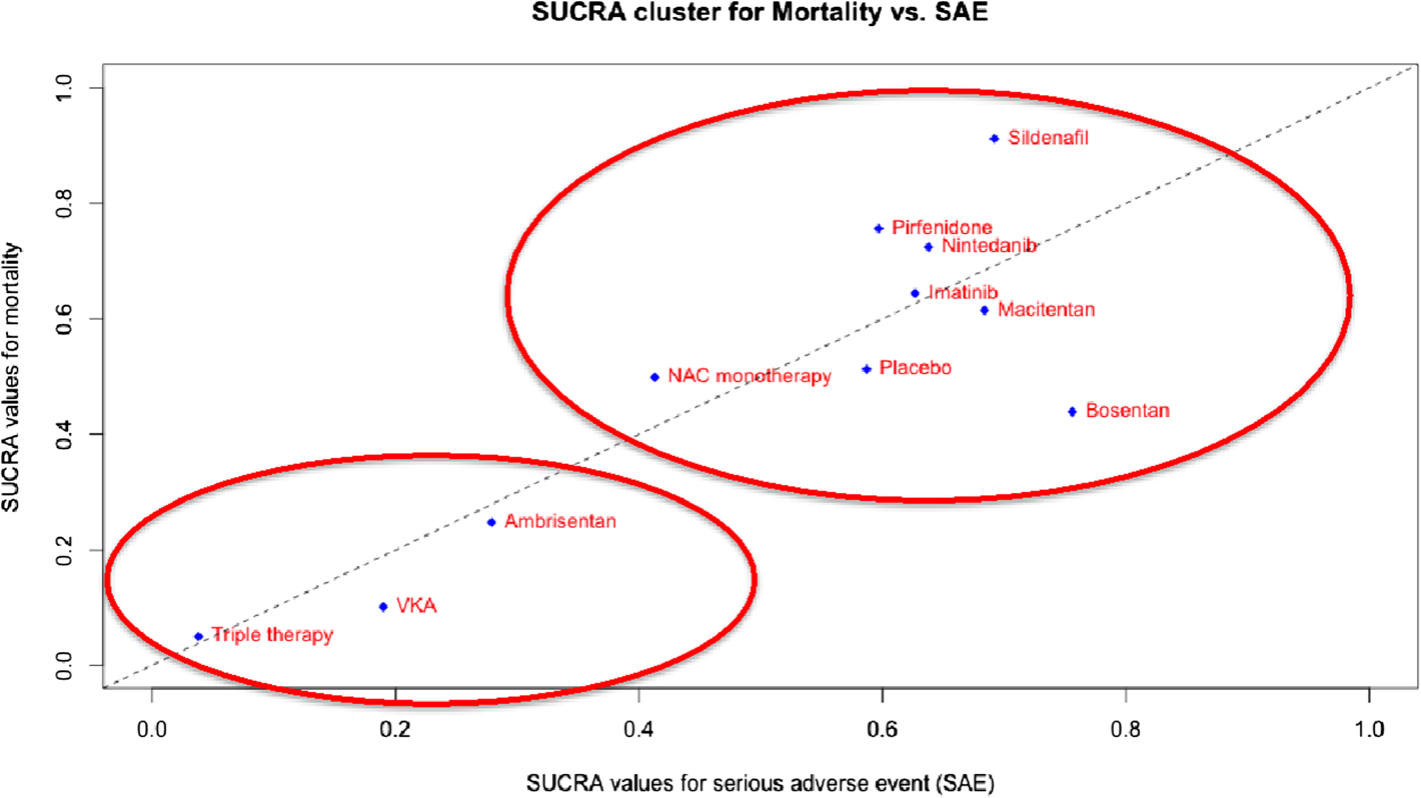
Scatterplot including surface under cumulative ranking curve (SUCRA) value for mortality on the y-axis and SUCRA value for severe adverse events (SAEs) on the x-axis. A higher SUCRA ranking for mortality indicates better survival whereas a higher SUCRA ranking for SAEs indicates fewer events associated with treatment. Cluster analysis demonstrates the division of treatments into two distinct groupings

Figure 2 shows a scatterplot including SUCRA value for mortality on the y-axis and SUCRA value for SAEs on the x-axis. Cluster analysis demonstrates the division of treatments into two distinct groupings. One cluster of interventions, which includes ambrisentan, triple therapy, and VKA, has lower SUCRA values for both outcomes compared with the other grouping.

## Discussion

The results of this NMA highlight potentially important differences in mortality and SAEs between different treatment interventions for IPF. Our findings suggest a possible mortality advantage of nintedanib, pirfenidone, and sildenafil compared to other treatments. Focusing on longer-term mortality data, by excluding the two trials of sildenafil with 6-month follow-up, we observed the potential survival benefit of nintedanib and pirfenidone compared to other treatment interventions. No significant difference was seen when comparing these two treatments to each other.

The strengths of this systematic review and NMA include the inclusion of RCTs that address a precise clinical question with well-defined IPF patients, focusing on outcomes that are important to patients. We conducted a comprehensive search and RoB assessment, with both processes involving duplicate review and third party adjudication if necessary. Using rigorous NMA methods [16], we used indirect evidence to compare the efficacy and safety profiles of active therapeutic agents investigated in patients with IPF, which allowed for assessment of comparative efficacy between IPF treatment interventions, providing the best estimates of effect. The GRADE approach also allowed reporting of the certainty in the evidence when interpreting each unique treatment comparison and across the network.

The benefits of any intervention must be weighed against potential harms or adverse effects. Although both pirfenidone and nintedanib are associated with SAEs, being primarily dermatologic manifestations and gastrointestinal disturbances, neither proved significantly worse than any other intervention. The SUCRA rankings for these interventions suggested that, although they were not likely to be the best options in terms of avoiding SAEs, they were not in the bottom of the rankings either. The balance between benefit and harm is demonstrated in Fig. 2 where treatments found in the upper right of the graph, such as nintedanib, pirfenidone, and sildenafil, are beneficial in terms of both mortality and SAE rates compared to other active interventions. The results further suggest that certain interventions for IPF, specifically triple therapy, VKAs, and ambrisentan, are associated with an increased risk of SAE with no demonstrated benefit.

The limitations of our review include the small number of studies relative to the number of comparisons considered, resulting in low certainty in estimates for many key comparisons. Although all included studies examined only IPF patients, there was also some heterogeneity in disease severity as assessed by PFTs, radiologic assessment, and follow-up time across studies. To incorporate heterogeneity in treatment effects, we employed random-effect assumptions. The subgroup analysis was also performed to examine the impact of including trials with shorter duration of follow-up. We were unable to perform the NMA of some other patient important outcomes such as quality of life indices, 6-minute walk test, or acute exacerbation rate due to the differential reporting of these outcomes across included studies and the relative inaccessibility of the primary data. Applying the NMA model to the limited number of studies that included these outcomes would lead to very imprecise and non-informative results. Therefore, it is possible that minimal important differences in treatment effects concerning other patient-important outcomes were missed [37].

## Conclusions

This NMA provides the best available estimates of treatment effect on overall mortality for IPF interventions combining all available evidence. It is the first analysis to provide comparative efficacy for patient important outcomes from interventions in IPF. Results suggest greater benefits of nintedanib and pirfenidone compared to other treatments, while no significant difference was seen when comparing these two interventions. Ambrisentan, VKA, and triple therapy are associated with harm and had no demonstrated benefit. However, given the limitations and low certainty in the evidence for most comparisons, conclusions should be interpreted with caution and clinical decision-making must be informed by the results of future head-to-head RCTs to confirm or refute these findings.

## Appendix

**Table 5:** Primary search strategy for MEDLINE

#	Search strategy
1	exp pulmonary fibrosis/
2	exp Fibrosis/
3	exp Respiratory Tract Diseases/
4	exp Respiratory System/
5	3 or 4
6	and 5
7	1 or 6 [PF Subject Headings]
8	((lung$ or respir$ or pulmonary or alveol$) adj6 (fibros$ or fibrotic or fibrous)).tw.
9	(Cystic adj fibro$).mp.
10	8 not 9 [Cystic Fibrosis articles eliminated]
11	(idiopa$ adj2 pulmonary adj2 fibro$).tw.
12	Idiopathic Pulmonary Fibrosis/
13	ipf.mp. and (idiopa$ or pulmonary).tw.
14	11 or 12 or 13 [Add IPF back in]
15	7 or 10 or 14 [PF Subject Headings -- Lung/Fibrosis Textword terms]
16	(interstitial$ adj3 (fibros$ or fibrotic or fibrous)).tw.
17	5 and 16 [Respiratory Subject Headings -- interstitial fibrosis Textword terms]
18	14 or 17 [PF Subject headings -- PF Textwords]
19	(((usual or ordinary) adj3 interstitial) and pneumo$).tw.
20	(cryptog$ and (fibros$ or fibrotic or fibrous) and alveol$).tw.
21	idiopa$ interstitial pneumoni$.tw.
22	19 or 20 or 21 [Other textwords used to describe IPF]
23	Lung Diseases, Interstitial/ or (interstitial adj lung adj disease$).tw. or (parenchymal.tw. and (lung$ or pulmonary)).tw.
24	(((unknow$ or uncertain$) adj4 (origin$ or cause$ or aetiol$ or etiol$)) or idiopa$).tw.
25	23 and 24 [Concept to describe idiopathic disease concept of ILD]
26	18 or 22 or 25 [PF Subject headings -- PF Textwords -- IPF Textwords]
27	limit 26 to human [must be indexed with human (animal could be included]
28	limit 26 to animal [must be indexed with animal (human could be included)]
29	26 not 27 not 28 [citations not indexed with human or animal]
30	27 or 29 [limited to human (may include animals) or citations without animal/human limits]
31	201404$.dp,ed,ep.
32	201405$.dp,ed,ep.
33	31 or 32
34	30 and 33
35	limit 34 to english language
36	limit 34 to abstracts
37	35 or 35

## Abbreviations

CrI: Credible interval
FVC: Forced vital capacity
GRADE: Grading of Recommendations Assessment, Development and Evaluation
IPF: Idiopathic pulmonary fibrosis
NMA: Network meta-analysis
OR: Odds ratio
PFT: Pulmonary function test
RCT: Randomized controlled trial
RoB: Risk of bias
SAE: Severe adverse events
SUCRA: Surface under the cumulative ranking
VKA: Vitamin K antagonist

## References

[1] Olson AL, Swigris JJ, Lezotte DC, Norris JM, Wilson CG, Brown KK (2007). Mortality from pulmonary fibrosis increased in the United States from 1992 to 2003. Am J Respir Crit Care Med.

[2] Raghu G, Collard HR, Egan JJ, Martinez FJ, Behr J, Brown KK (2011). An official ATS/ERS/JRS/ALAT statement: idiopathic pulmonary fibrosis: evidence-based guidelines for diagnosis and management. Am J Respir Crit Care Med.

[3] Martinez FJ, de Andrade JA, Anstrom KJ, King TE, Raghu G (2014). Randomized trial of acetylcysteine in idiopathic pulmonary fibrosis. N Engl J Med.

[4] King TE, Bradford WZ, Castro-Bernardini S, Fagan EA, Glaspole I, Glassberg MK (2014). A phase 3 trial of pirfenidone in patients with idiopathic pulmonary fibrosis. N Engl J Med.

[5] Richeldi L, du Bois RM, Raghu G, Azuma A, Brown KK, Costabel U (2014). Efficacy and safety of nintedanib in idiopathic pulmonary fibrosis. N Engl J Med.

[6] Atkins CP, Loke YK, Wilson AM (2014). Outcomes in idiopathic pulmonary fibrosis: a meta-analysis from placebo controlled trials. Respir Med.

[7] Bajwa EK, Ayas NT, Schulzer M, Mak E, Ryu JH, Malhotra A (2005). Interferon-gamma1b therapy in idiopathic pulmonary fibrosis: a metaanalysis. Chest.

[8] Loveman E, Copley VR, Colquitt JL, Scott DA, Clegg AJ, Jones J (2014). The effectiveness and cost-effectiveness of treatments for idiopathic pulmonary fibrosis: systematic review, network meta-analysis and health economic evaluation. BMC Pharmacol Toxicol..

[9] Raghu G, Collard HR, Anstrom KJ, Flaherty KR, Fleming TR, King TE (2012). Idiopathic pulmonary fibrosis: clinically meaningful primary endpoints in phase 3 clinical trials. Am J Respir Crit Care Med.

[10] Raghu G, Rochwerg B, Zhang Y, Garcia CA, Azuma A, Behr J (2015). An official ATS/ERS/JRS/ALAT clinical practice guideline: treatment of idiopathic pulmonary fibrosis. An update of the 2011 Clinical Practice Guideline. Am J Respir Crit Care Med.

[11] Higgins JP, Altman DG, Gotzsche PC, Juni P, Moher D, Oxman AD (2011). The Cochrane Collaboration’s tool for assessing risk of bias in randomised trials. BMJ (Clinical research ed).

[12] Evidence Partners. http://distillercer.com/resources/methodological-resources/risk-of-bias-commentary/. Accessed date 7 Apr 2015.

[13] Jansen JP, Cope S (2012). Meta-regression models to address heterogeneity and inconsistency in network meta-analysis of survival outcomes. BMC Med Res Methodol..

[14] Jansen JP, Naci H (2013). Is network meta-analysis as valid as standard pairwise meta-analysis? It all depends on the distribution of effect modifiers. BMC Med.

[15] Higgins JP, Thompson SG (2002). Quantifying heterogeneity in a meta-analysis. Stat Med.

[16] Mills EJ, Ioannidis JP, Thorlund K, Schunemann HJ, Puhan MA, Guyatt GH (2012). How to use an article reporting a multiple treatment comparison meta-analysis. JAMA.

[17] Gelman ARD (1992). Inferences from iterative simulation using multiple sequences. Stat Sci..

[18] Salanti G, Ades AE, Ioannidis JP (2011). Graphical methods and numerical summaries for presenting results from multiple-treatment meta-analysis: an overview and tutorial. J Clin Epidemiol.

[19] van Valkenhoef G, Tervonen T, Zhao J, de Brock B, Hillege HL, Postmus D (2012). Multicriteria benefit-risk assessment using network meta-analysis. J Clin Epidemiol.

[20] Puhan MA, Schunemann HJ, Murad MH, Li T, Brignardello-Petersen R, Singh JA (2014). A GRADE Working Group approach for rating the quality of treatment effect estimates from network meta-analysis. BMJ..

[21] Noth I, Anstrom KJ, Calvert SB, De Andrade J, Flaherty KR, Glazer C (2012). A placebo-controlled randomized trial of warfarin in idiopathic pulmonary fibrosis. Am J Respir Crit Care Med.

[22] Raghu G, Anstrom KJ, King TE, Lasky JA, Martinez FJ (2012). Prednisone, azathioprine, and N-acetylcysteine for pulmonary fibrosis. N Engl J Med.

[23] Jackson RM, Glassberg MK, Ramos CF, Bejarano PA, Butrous G, Gomez-Marin O (2010). Sildenafil therapy and exercise tolerance in idiopathic pulmonary fibrosis. Lung.

[24] Zisman DA, Schwarz M, Anstrom KJ, Collard HR, Flaherty KR, Hunninghake GW (2010). A controlled trial of sildenafil in advanced idiopathic pulmonary fibrosis. N Engl J Med.

[25] Homma S, Azuma A, Taniguchi H, Ogura T, Mochiduki Y, Sugiyama Y (2012). Efficacy of inhaled N-acetylcysteine monotherapy in patients with early stage idiopathic pulmonary fibrosis. Respirology (Carlton, Vic).

[26] Tomioka H, Kuwata Y, Imanaka K, Hashimoto K, Ohnishi H, Tada K (2005). A pilot study of aerosolized N-acetylcysteine for idiopathic pulmonary fibrosis. Respirology (Carlton, Vic).

[27] Azuma A, Nukiwa T, Tsuboi E, Suga M, Abe S, Nakata K (2005). Double-blind, placebo-controlled trial of pirfenidone in patients with idiopathic pulmonary fibrosis. Am J Respir Crit Care Med.

[28] Noble PW, Albera C, Bradford WZ, Costabel U, Glassberg MK, Kardatzke D (2011). Pirfenidone in patients with idiopathic pulmonary fibrosis (CAPACITY): two randomised trials. Lancet.

[29] Taniguchi H, Ebina M, Kondoh Y, Ogura T, Azuma A, Suga M (2010). Pirfenidone in idiopathic pulmonary fibrosis. Eur Respir J.

[30] King TE, Behr J, Brown KK, du Bois RM, Lancaster L, de Andrade JA (2008). BUILD-1: a randomized placebo-controlled trial of bosentan in idiopathic pulmonary fibrosis. Am J Respir Crit Care Med.

[31] King TE, Brown KK, Raghu G, du Bois RM, Lynch DA, Martinez F (2011). BUILD-3: a randomized, controlled trial of bosentan in idiopathic pulmonary fibrosis. Am J Respir Crit Care Med.

[32] Raghu G, Behr J, Brown KK, Egan JJ, Kawut SM, Flaherty KR (2013). Treatment of idiopathic pulmonary fibrosis with ambrisentan: a parallel, randomized trial. Ann Intern Med.

[33] Raghu G, Million-Rousseau R, Morganti A, Perchenet L, Behr J (2013). Macitentan for the treatment of idiopathic pulmonary fibrosis: the randomised controlled MUSIC trial. Eur Respir J.

[34] Daniels CE, Lasky JA, Limper AH, Mieras K, Gabor E, Schroeder DR (2010). Imatinib treatment for idiopathic pulmonary fibrosis: Randomized placebo-controlled trial results. Am J Respir Crit Care Med.

[35] Richeldi L, Costabel U, Selman M, Kim DS, Hansell DM, Nicholson AG (2011). Efficacy of a tyrosine kinase inhibitor in idiopathic pulmonary fibrosis. N Engl J Med.

[36] Demedts M, Behr J, Buhl R, Costabel U, Dekhuijzen R, Jansen HM (2005). High-dose acetylcysteine in idiopathic pulmonary fibrosis. N Engl J Med.

[37] Schunemann HJ, Guyatt GH (2005). Commentary--goodbye M(C)ID! Hello MID, where do you come from?. Health Serv Res.

